# Winter warming is ecologically more relevant than summer warming in a cool-temperate grassland

**DOI:** 10.1038/s41598-019-51221-w

**Published:** 2019-10-10

**Authors:** Juergen Kreyling, Kerstin Grant, Verena Hammerl, Mohammed A. S. Arfin-Khan, Andrey V. Malyshev, Josep Peñuelas, Karin Pritsch, Jordi Sardans, Michael Schloter, Jan Schuerings, Anke Jentsch, Carl Beierkuhnlein

**Affiliations:** 1 Greifswald University, Institute of Botany and Landscape Ecology, Experimental Plant Ecology, Soldmannstraße 15, D-17487 Greifswald, Germany; 2 University of Bayreuth, BayCEER, Disturbance Ecology, D-95440 Bayreuth, Germany; 3 Helmholtz Zentrum München, Research Unit Comparative Microbiome Analysis, Ingolstädter Landstr. 1, 85764 Oberschleissheim, Germany; 4 Department of Forestry and Environmental Science, Shahjalal University of Science and Technology, Sylhet, 3114 Bangladesh; 5 CSIC, Global Ecology Unit CREAF-CSIC-UAB, Bellaterra, Catalonia, 08193 Spain; 6 CREAF, Cerdanyola del Vallès, Catalonia, 08193 Spain; 7 Helmholtz Zentrum München, Institute of Biochemical Plant Pathology, Ingolstaedter Landstr. 1, 85764 Oberschleißheim, Germany; 8 University of Bayreuth, BayCEER, Biogeography, D-95440 Bayreuth, Germany

**Keywords:** Climate-change ecology, Climate-change ecology

## Abstract

Climate change affects all seasons, but warming is more pronounced in winter than summer at mid- and high latitudes. Winter warming can have profound ecological effects, which are rarely compared to the effects of summer warming, and causal explanations are not well established. We compared mild aboveground infrared warming in winter to warming in summer in a semi-natural, cool-temperate grassland in Germany for four years. Aboveground plant biomass increased following winter warming (+18%) and was unaffected by summer warming. Winter warming affected the composition of the plant community more than summer warming, favoring productive species. Winter warming increased soil respiration more than summer warming. Prolonged growing seasons and changes in plant-community composition accounted for the increased aboveground biomass production. Winter warming stimulated ecological processes, despite causing frost damage to plant roots and microorganisms during an extremely cold period when warming reduced the thermal insulation provided by snow. Future warming beyond such intermittent frosts may therefore further increase the accelerating effects of winter warming on ecological processes.

## Introduction

Winter warming is projected to outpace summer warming by 2 °C in central Europe by 2071–2100, with even larger differences farther north (RCP 8.5^1,2^). A solid understanding of the relevant winter processes and their ecological importance is still lacking, because manipulation experiments simulating climate change commonly apply uniform warming only during the growing season^3,4^. Predicting the ecological effects of seasonally non-uniform warming is consequently highly uncertain^5^.

Summer warming can increase aboveground plant biomass production, but this effect is often limited by water, light, and nutrient availability^3^. Plants in areas characterized by seasonal frost are directly limited by temperature in winter^6^, and winter warming consequently increases the spring^7^ or annual^8^ aboveground plant biomass production in temperate grasslands, particularly if warming leads to the absence of soil frost^9^. Seasonal warming should further induce changes in community composition due to species-specific physiological adaptations to seasonal variation in climatic conditions^10^. Key ecosystem processes such as plant biomass production and decomposition are affected by plant-community composition^11,12^. Furthermore, the sensitivity of soil respiration to temperature is higher in winter and spring than summer and autumn^13,14^. Finally, the ecological sensitivity toward winter warming is probably linked to the trade-off between the length of the growing season and frost damage. Warming in late winter advances spring plant phenology^15^ and thereby lengthens the growing season and enhances aboveground biomass production. This earlier start of the growing season due to warming, however, can also lead to pre-mature de-hardening, with an increased risk of subsequent frost damage^16^. Snow cover plays a crucial role in ecosystem processes in colder climates^17^, but winter warming is decreasing snow cover and depth in cool and cold temperate ecosystems^18,19^. Snow, however, is an excellent insulator, and reduced snow cover and depth can lead to “colder soils in a warmer world”^20^ because of the reduced insulation of the soil during atmospheric frost. The effect of this insulation complicates predictions of soil biotic responses to winter warming. Soil warming generally increases soil biotic activity^21^, N mineralization and availability^22^, and soil respiration^3^ in temperate ecosystems. Reduced snow cover can thus decrease soil biotic activity despite warming^23–25^. Reduced snow cover and increased soil freezing may consequently offset any stimulating effect of warming, leading to no net effects on important ecosystem processes such as litter decomposition^26,27^. Winter warming can lengthen the growing season by later senescence in autumn and earlier green-up in spring but can also increase frost damage of plants and soil biota^28^.

Taken together, winter warming can lengthen the growing season^1,15^ but can also result in increased frost damage of plants and soil biota due to reduced frost hardening^16^ and due to reduced insulation by snow^20,28^. Thus, detailed observations of plant performance and soil biotic activity during winter warming are needed for a better mechanistic understanding of the season-specific effects of warming^17^. Here, we present results from a warming experiment over four consecutive years in which summer warming (April to September), winter warming (October to March), and ambient reference conditions were compared in a semi-natural temperate grassland. These permanent grasslands are used for hay production with 2–5 cuts per year or as pasture since medieval times and harbor substantial amounts of native species on 13% of the landscape in Europe^29^. We hypothesized that winter warming would increase annual aboveground biomass production and soil respiration stronger than summer warming and furthermore has a greater impact on plant species composition. In order to explain the observed net effects of winter warming (annual aboveground biomass production, plant community composition, soil respiration), plant performance (greenness, root growth, leaf C:N ratio) and soil biotic processes (microbial biomass, potential extracellular enzyme activity, N-availability, soil respiration) were quantified during winter.

## Results

**Figure 1:**
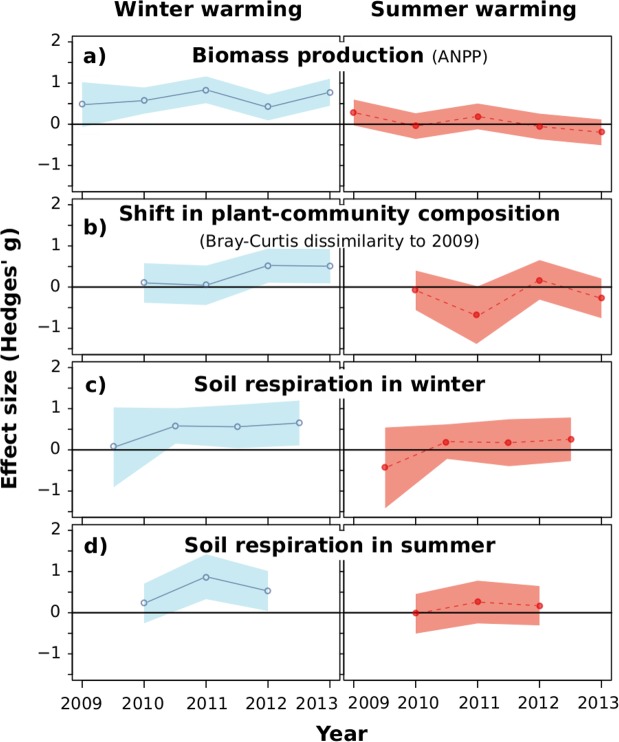
Winter warming is ecologically more relevant than summer warming. (**a**) Aboveground net primary production (ANPP, sum of two destructive harvests of 0.2 m² y^−1^), (**b**) changes in plant-community composition per plot compared to its initial composition in 2009 expressed as Bray-Curtis distance (based on estimates of species-specific cover (1 m²) in June), and (**c**,**d**) soil respiration (mean of monthly measurements separated by winter (**c**) and summer (**d**) for the entire study period). The effect sizes as compared to reference conditions are displayed as Hedges’ g (n = 10) per sampling date and treatment and its 95% confidence intervals. A treatment is considered significant if the confidence band does not include zero (gray horizontal line). Note that the year 2009 displays pre-treatment conditions.

Aboveground infrared warming in winter led to 0.6 and 1.7 °C warming of air and soil, respectively, from October to March. Warming in summer increased air and soil temperature by 1.7 and 0.6 °C, respectively, from April to September (Supplementary Information Fig. 1) over four consecutive years. Winter warming had larger effects than summer warming on the ecological parameters measured. Aboveground net primary production (ANPP, i.e. the biomass produced over one year) was on average 18% higher and differed significantly from control in all four study years, with increasing effect sizes over time, in the winter-warming treatment compared to reference conditions (Fig. 1a). Summer warming did not significantly affect ANPP in any year. Annual soil respiration throughout the study period increased by 9.3% due to winter warming and by 5.9% due to summer warming. Winter warming increased soil respiration in both winter and summer, with effects being significant from the second warming campaign in both seasons (Fig. 1c,d), but summer warming increased soil respiration only in summer, again with significant effects from the second summer warming campaign onwards. Finally, winter warming affected plant-community composition more than summer warming over time with significant differences to control in the third and fourth study year while summer warming did not significantly affect plant-community composition (Fig. 1b). As indicated by an Indicator Species Analyses, this shift in community composition was in favor of tall and productive species in the winter warming treatment while no such clear trend was found in the summer warming treatment (Supplementary Information Table 1).

**Figure 2:**
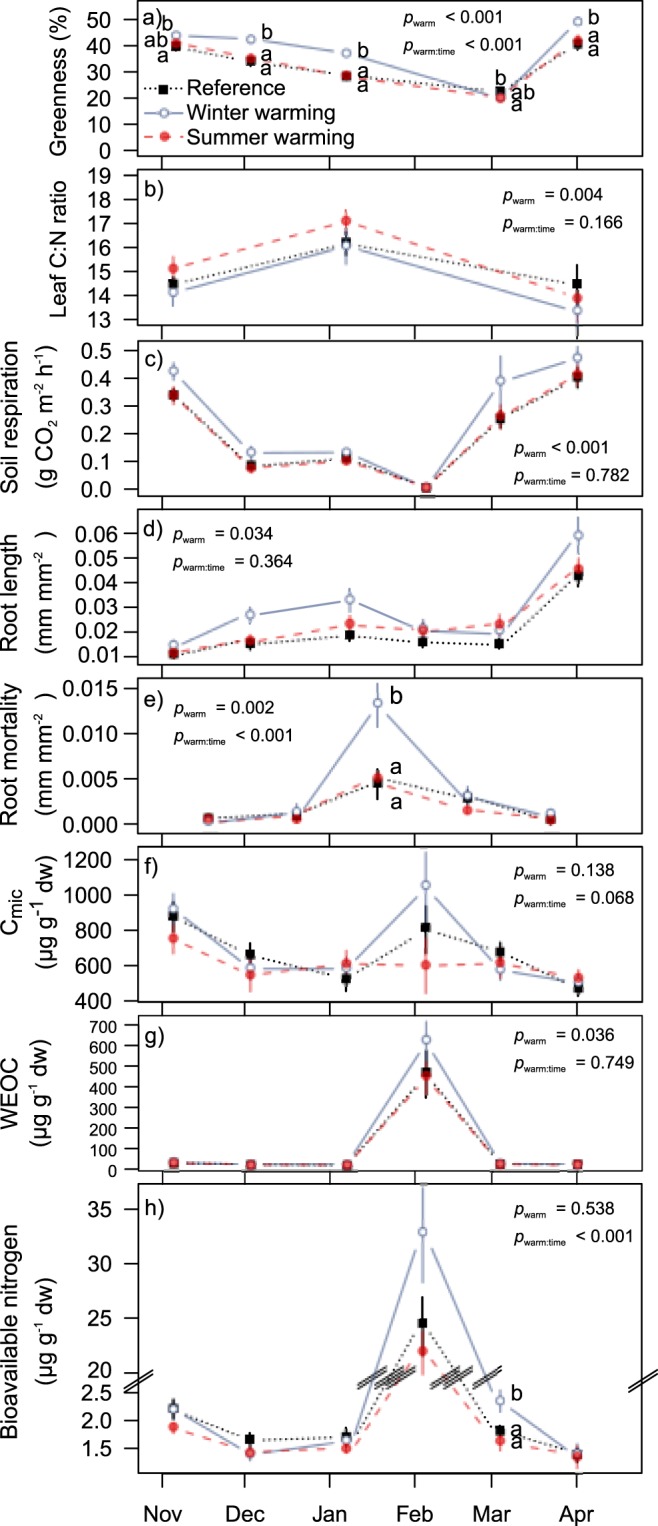
Responses to warming treatments in winter 2011/12 (active warming for winter warming but no warming for the summer-warming treatment during this period). Greenness at plot level was not measured in February due to snow cover. Leaf C:N was measured for leaves of the same three species (*Alopecurus pratensis*, *Plantago lanceolata*, and *Rumex acetosa*) in each plot. Root mortality is the sum of roots dying between two samplings. C_mic_, microbial biomass; WEOC, water-extractable organic C. Means ± SEMs (n = 10) per sampling date and treatment (with the three species per plot as nested replicates for leaf C:N) are displayed. Lowercase letters indicate homogeneous groups per date based on Tukey post hoc tests (only tested if the interaction between warming and time was significant and displayed for dates that differed among the treatment levels in the post hoc test).

The increase in aboveground biomass production and soil respiration due to winter warming may have been due to the change in plant-community composition, but also to other winter ecological processes such as plant performance (greenness, root length and mortality, and leaf C:N ratio) and soil biotic processes (soil respiration, microbial biomass, N availability, and potential extracellular enzymatic activity) (Fig. 2 and Supplementary Information Fig. 2). The intensive winter sampling campaign indicated that winter warming lengthened the growing season both in late autumn and in early spring, indicated by plant greenness, root length, and soil respiration (Fig. 2a–c). Lower leaf C:N ratios (Fig. 2d) further indicated active N uptake by plant roots late in autumn and early in spring, supporting the conclusion that winter warming stimulated aboveground plant biomass production during these periods.

A natural cold period when winter warming had melted the snow cover, led to colder minimum temperatures during our intensive winter sampling than under the reference conditions (minimum air temperature at plant height, −14.0 versus −9.7 °C; minimum soil temperature, −4.0 versus −2.2 °C; Supplementary Information Fig. 3). This result supports the notion that winter warming can lead to colder soils if it reduces snow cover^20^. This frost was the most likely cause of the decrease in greenness (Fig. 2a), the increase in root mortality, and the peaks in microbial biomass (C_mic_), water-extractable organic C (WEOC), and bioavailable N (Fig. 2e–h) in the winter-warming treatment, because no other abiotic parameter reached stressful levels during this time (radiation: PAR < 800 µmol m^−2^ s^−1^; soil moisture well above the wilting point of 8%).

## Discussion

Winter warming was ecologically more important than summer warming in our seasonal warming experiment in a permanent temperate grassland over four consecutive years. Species differ in their potential to profit from a prolonged growing season^10^. Accordingly, winter warming in our study was accompanied by changes in plant-community composition toward more productive species (Supplementary Information Table 1). More productive (taller) species may benefit more from a longer growing season and thereby become more dominant by outcompeting smaller, light demanding species by shading. Changes in plant-community composition induce lasting changes in ecosystem functioning, relevant not only for aboveground biomass production, but also for decomposition and nutrient cycling^12^ or N leaching^30^ and potentially leading to hysteresis and regime shifts in plant-community composition and ecosystem functions^31^. Changes in plant-community composition are therefore more relevant than short-term physiological effects, which may simply be due to phenotypic plasticity^32^, are generally reversible and may be transient. The increase in more productive species may further lead to a decrease in short but light-demanding species in the long term. In addition to management effects or eutrophication, winter warming may therefore play a role, which has been overlooked, in the biodiversity decline^33^ of protected (European Habitats Directives) lowland permanent grasslands.

A reduction in snow cover and the loss of its insulating effect followed by subsequent frost can increase frost damage^30,34,35^. Frost tolerance differs among grass species^36^ and can therefore be expected to act as a selective driver that can contribute to changes in community composition. Cold acclimation in microbes is accompanied by net N mineralization^37^, but the parallel peak in WEOC indicates microbial death rather than dormancy^37,38^. In addition to the lysis of microbial cells and roots, soil-bound organic C and other nutrients become accessible by physical stress that fragments soil aggregates^39^. These easily accessible nutrients induce high rates of microbial growth and activities immediately after physical stress^40^ or with some delay^41^, in line with the peak in microbial biomass after the frost in our study.

The natural cold period during our intensive winter sampling campaign decreased the minimum soil and air temperatures and presumably induced frost damage in plants and microorganisms in the winter-warming treatment, but winter warming still increased ANPP during the growing season following the frost. For future projections, this result implies that the effects of winter warming will even further increase their effects on ANPP and soil respiration, because such cold periods may eventually become less frequent in an increasingly warming environment^1,19^.

We focused on separating the effects of summer and winter warming and accounting for the latter. Warming will likely occur throughout the year, even though its magnitude may differ among seasons^1,5^. Combined warming effects, particularly on opposing trends such as the changes in plant-community composition, are difficult to predict and require further research that includes seasonally non-uniform warming lasting long enough for studying community changes and acclimation effects in plants and microorganisms^42,43^. Our soil warming of 1.6 °C was at the lower end of commonly applied warming manipulations (1.5 to 6 °C^3^). The clear and strong ecological net effects (ANPP, plant-community composition, and soil respiration) in this study are therefore even more relevant and emphasize the ecological importance of highly probable winter warming. This is even more remarkable as other warming experiments report high inertia in grassland community composition with changes only after seven years of year-round warming^44^. We hypothesize that the comparably quick response to winter warming in terms of community composition in our experiment points towards a high vulnerability to warming of cool-temperate ecosystems which formerly showed regular protective snow cover over winter.

Our results emphasize the importance of the seasonality of warming. Mild winter warming was ecologically more relevant than mild summer warming for a cool temperate ecosystem. Our data suggest that this difference is due to the effects of winter warming lengthening the growing season and changing plant-community compositions toward more productive species. Frost damage may mitigate the effects of winter warming, because the reduction in snow cover led to frost damage during natural cold periods. Such soil frosts due to reduced snow cover, however, are expected to only be transient for cool temperate ecosystems. Future warming will therefore likely further increase the stimulation of ecosystem processes mainly by winter warming.

## Methods

### Study site

This study was conducted in the Ecological-Botanical Garden of the University of Bayreuth, Germany (49°55′19″N, 11°34′55″E; 365 m a.s.l.). The regional climate is temperate and moderately continental (mean annual air temperature of 8.2 °C and mean annual precipitation of 724 mm for 1971–2000; data from the German Weather Service). Mean winter air temperature (October-March) is 3.0 °C, and mean summer air temperature (April-September) is 14.4 °C. The site was covered by snow on an average of 36 d per winter (2009–2014). The soil is a Gleysol. A 30-cm Ap horizon (42% sand, 43% silt, 15% clay) overlays a clayey Bg horizon. The main rooting zone is within the upper 15 cm, and few roots penetrate the Bg horizon. The mean pH of the topsoil is 4.1 (1 M KCl). The experimental site is a semi-natural grassland that has not been plowed or fertilized for at least 25 years. The site was mown twice a year for hay production prior to the start of the experiment, leading to disturbance-tolerant, species-rich, perennial semi-natural grassland communities typical of large areas throughout Europe^29^ and dominated by tall grasses such as *Alopecurus pratensis* L. (meadow foxtail) and *Arrhenatherum elatius* (L.) P.Beauv. ex J.Presl & C.Presl (tall oat-grass). The most common herb species include *Plantago lanceolata* L. (ribwort plantain), *Rumex acetosa* L. (common sorrel), and *Cerastium fontanum* subsp*. vulgare* (Hartm.) Greuter & Burdet (mouse ear chickweed), and the most abundant legume is *Trifolium pratense* L. (red clover). One square meter contains an average of 14 species. All plants are C3 species.

### Experimental design

The field experiment was initially carried out in a fully crossed two-factorial design manipulating (1) temperature (ambient, winter warming, summer warming) and (2) variability of intra-annual precipitation. We focused on seasonal warming to identify its relative net effects (compared to no warming) using detailed winter measurements of plant performance and soil biotic activity. We collected data from two precipitation treatments (low and high precipitation variability, with the latter including an early summer drought, i.e. 42 d without rainfall, combined with heavy rainfall, i.e. adding the missing amount due to the drought treatment within 2 d, both treatments totaling the same annual precipitation) and accounted for the potential effects of the precipitation treatment by adding it as a random effect in the statistical models (see below and Grant *et al*.^4^). Our results were thus generalized to different regimes of growing-season precipitation, which did not interact significantly with the seasonal warming treatment, based on pre-tests with summer precipitation as an additional fixed factor. The design consisted of 30 plots, each 1.5 × 1.5 m, with 10 replicates of each warming treatment (five true replicates of the two precipitation treatments). The warming-treatment plots were blocked within each precipitation treatment, with their position per block assigned randomly.

Temperature was manipulated either during the winter (October-March) or summer (April-September) starting in October 2009. The temperature was increased using overhead infrared heating lamps equipped with reflector domes (IOT/90 250 W, Elstein-Werk M. Steinmetz GmbH & Co. KG, Northeim, Germany) at a height of 0.8 m, theoretically delivering 60 W per plot. The lamps were raised to 1 m when tall grasses reached a height of 0.8 m later in the growing season and were lowered again to 80 cm after the harvest. Ambient plots were equipped with dummy heaters. The spatial homogeneity of the warming was consistently high within the area (1 m²) used to sample all response parameters (ΔT_air(+5cm)_ = 0.2 °C and ΔT_soil(−2cm)_ = 0.3 °C between five sensors installed at 0, 25, and 50 cm from the center of the plot during winter 2009/2010, and ΔT_air(+5cm)_ = 0.1 °C and ΔT_soil(−2cm)_ = 0.3 °C between three sensors installed at 0, 37.5, and 75 cm from the center of the plot during summer 2011). Snow depth per plot was measured manually by a yard stick at all days with snow on the ground. Air temperature at plant height within the plot (+5 cm) was measured by thermistors (B57863-106 S302-F40, EPCOS AG, München, Germany) with 10 radiation shields. Soil temperature at −2 cm was measured by the same sensors used for measuring air temperature but without shields (n = 10). Soil moisture was measured by 30 FD-sensors ECH2O (Decagon Devices, Pullman, USA) at −2 to −7 cm. All data were collected from the center of each plot directly below the lamp to avoid possible edge effects created by temperature gradients with increasing distance from the lamp.

### Response parameters

Annual net effects of the three warming treatments on aboveground biomass production, plant-community composition, and soil respiration were analyzed throughout the study period (one pre-treatment year and four years of warming). Intensive winter sampling, focusing on plant and soil biotic activity during one winter, was conducted to obtain a better mechanistic understanding of the effects of winter warming.

#### Annual net effects

Aboveground biomass was destructively harvested twice a year (June and September). Destructive harvests are part of the disturbance regimes of these semi-natural systems, and the frequency, timing, and intensity of our harvests resembled local agricultural routines for an extensively used grassland. For each harvest, a steel frame (0.1 m²) was placed twice in the central part of each plot, so that two samples of plant material per plot could be collected. All plant material was cut 3 cm above the soil surface within the steel frame. One sample was sorted into functional groups (forbs, graminoids, and legumes), and the other sample was sorted into species. All plant material was dried to a constant weight at 60 °C and then weighed (Ohaus NavigatorTM, Ohaus Corporation, Parsippany, USA; accuracy ±0.01 g). Total annual aboveground net primary productivity (ANPP) was calculated as the total biomass of all plant samples within each plot for each year based on the sampled area of 0.2 m². The entire plot was then mown to +3 cm.

Species-specific cover was estimated visually on a continuous scale immediately before each summer harvest and included all species within the central 1 m² of each plot. The same two observers working together estimated all covers. We used these data to determine the changes in community composition, quantified as the compositional changes in each plot, comparing its pattern of species abundance to its initial pattern (before the start of the experimental warming) at each time step using Bray-Curtis dissimilarity^45^. Non-random occurrence of particular species among the warming treatments was tested by an analysis of indicator species^46^. The statistical significance of preferential occurrence was evaluated using a randomization procedure with 1000 permutations^46^. No species preferentially occurred in 2009, the pre-treatment year. The results for these response parameters were qualitatively similar whether species-specific biomass data or cover data were used. We report the cover data, because this data set includes more species due to the larger spatial scale (1 versus 0.1 m²).

Soil respiration was measured in each plot with a respiration chamber connected to a non-dispersive infrared gas analyzer (SPC-1 & EGM-4, PP-systems, Amesbury, USA). The respiration chamber was placed on PVC collars to close the system. The collars (10 cm in diameter, 5 cm in height) were installed 4 cm into the soil one month before the start of measurements. The collars were elongated by open adaptor tubes to conduct measurements above the snow if snow was forecasted. All aboveground plant material was clipped from the collar the day before each measurement. Measurements were conducted monthly but with weekly or biweekly measurements during some campaigns. CO_2_ fluxes were measured for 4 min to reach a stable flux, and the average of the final four flux values was used for the analyses.

#### Intensive winter sampling

A set of response parameters of plant and soil biotic activity was quantified during winter 2011/2012 to identify the processes leading to changes in the net effects described above in response to winter warming. Samples were collected each month during the period of winter warming (October-March, with a final sampling in early April).

Potential changes in the length of the growing season were quantified by measuring the greenness of the plots. Greenness is directly correlated with photosynthetic potential^47^, so we used it as a surrogate for potential aboveground plant activity. Plot greenness was quantified monthly using digital photographs taken under standardized light conditions. A portable light-tight box (56 × 55 × 75 cm) with a camera (Nikon D2x, Nikon Corporation, Tokyo, Japan) and artificial lighting (two flashes) was used. The calculation of greenness was based on Marchand *et al*.^47^, using a transformation from RGB pictures to the HSL color space. The threshold for the ‘greenness’ of the hue band was determined using manually calibrated/optimized reference lookup tables for all bands implemented as lookup images. The percentage of greenness was calculated and processed with the same lookup tables and same parameters for all pictures and all time steps using ImageMagick 6.7.6–5 (ImageMagick Studio LLC, Landenberg, USA). Consequently, our ‘greenness’ is a relative measure comparable among all pictures in our study, expressed as the relative number of green pixels, i.e. the photosynthetic potential, of the plant community. The relative differences among the treatments were especially important, because they indicated alterations from natural seasonal patterns of greenness.Greenness could not be measured in February because snow covered the plots.

Leaf C and N concentrations were measured repeatedly to determine if the plants were taking up N over winter, which could have been mineralized by increased soil biotic activity (see below for quantification) or lysed by soil biota due to frost damage. Changes in leaf C:N can (1) indicate plant activity, (2) account for the fate of available N in the soil, and (3) help to account for the higher aboveground plant biomass production during the growing season. Soil CN ratio was 11.8 ± 0.2 SE in summer 2011. Leaf C and N were determined monthly for mixed samples of three leaves from each of the three most frequent species (40, 4, and 3% mean covers per plot in summer 2012 for *Alopecurus pratensis*, *Plantago lanceolata*, and *Rumex acetosa*, respectively). The leaves were oven-dried at 70 °C for 72 h, pulverized, re-dried at 70 °C for 48 h, and stored in desiccators until analyzed (<15 d). Thereafter, 0.7–1.5 mg of the dried and pulverized samples were weighed with a Mettler Toledo MX5 microbalance, and the concentrations of the elements were determined by combustion coupled to gas chromatography using an Elemental Analyzer CHNS Eurovector 3011 Thermo Electron Gas Chromatograph, model NA 2100 (CE Instruments/Thermo Electron, Milan, Italy).

Fine roots are essential for taking up water and nutrients. Differences in winter root growth or mortality can provide information on frost damage or increased belowground plant activity, which can affect the production of annual aboveground biomass^48^. Root length at each sampling date and root mortality between the sampling dates were determined monthly using a minirhizotron technique. A clear plastic tube (5 cm diameter) was installed at 45° to a depth of 45 cm in each plot in 2008. The aboveground sections of the tubes were covered with adhesive aluminum foil, and the tubes were capped to prevent the entry of water, dust, light, and heat. The roots were scanned with a root scanner adapted from an ordinary computer scanner (Optic slim 2400+) mounted on a metal pole that was rotated by an electric motor. The scans (18 × 21.6 cm) were at an angle of about 300°. Root lengths for the entire scan were quantified for each tube using Rootfly (Rootfly Development Team, Version 2.0.2, GNU General Public License). Root mortality was calculated as the length of roots dying between the sampling dates.

Soil microbiotas are essential for decomposing organic matter and for soil respiration. C_mic_ was measured by chloroform-fumigation extraction^49^ using 5 g of fresh soil (three technical replicates per plot collected using a stainless-steel corer to −10 cm) within 2 d after sampling. Samples in glass vials were placed in a desiccator containing 25 mL of ethanol-free chloroform (Merck KGaA, Darmstadt, Germany) and then extracted with 20 mL of 0.01 M CaCl_2_ (1:4 extraction ratio) in a rotary shaker for 30 min. The soil suspension was then filtered with a 595 1/2 paper filter (Whatman International LTD, VWR International GmbH, Darmstadt, Germany) and stored at −20 °C until analysis. Non-fumigated samples served as controls to assess the amount of WEOC. WEOC and microbial biomass were measured using a total organic-C analyzer DIMATOC 2000 (DIMATEC Analysentechnik GmbH, Essen, Germany) by catalytic high-temperature oxidation. C_mic_ was calculated using the method described by Joergensen and Mueller^50^: C_mic_ = EC/KEC, where EC is the difference between the C extracted from the fumigated and nonfumigated samples, and KEC = 0.41. Non-fumigated extracts were also used to measure water-extractable N, which was considered as the bioavailable fraction (tNb).

### Statistical analyses

The ecological relevance of seasonal warming throughout the study period was determined by calculating Hedges’ d effect sizes for winter and summer warming and comparing them to the reference. A treatment was considered to have a significantly positive effect if the 95% confidence intervals of the mean effect size did not include values <0 and was considered to have a significantly negative effect if the intervals did not include values >0^51^. The analyses used the R ‘effsize’ package, version 0.7.1. These results were supported by linear mixed-effects models (see below), which qualitatively produced the same insights.

For the detailed winter-sampling campaign, we constructed linear mixed-effect models in combination with analyses of variance (ANOVAs) to test for effects of the warming manipulations on each response variable. The blocked design was taken into account by a random factor describing the spatial configuration of the experiment (row/column) in the model^52^. The repeated measurements over time were also taken into account by adding the plot ID as a random effect. Fixed effects included the warming treatment and its interaction with time. Residual versus fitted plots and qq-plots based on the model were checked for homogenous variance and normal distributions of the residuals to validate the linear mixed-effect models^52^. The response parameters for which the parametric assumptions were not met were transformed as: rank (leaf C:N), log(1 + soil respiration), rank(root length), log(1 + tNb). For leaf C:N, species identity was added as an additional random effect if the initial models failed to identify significant interactions between warming treatment and species identity. The significance level for all tests was set to 0.05. All statistical analyses were performed using R 3.3.2^53^. The ‘lmerTest’ package, version 2.0–33, and the ‘emmeans’ package, version 1.1, were used for the linear mixed-effect models and multiple post-hoc comparisons, respectively. The analysis of indicator species used the ‘labdsv’ package, version 1.8-0.

## Supplementary information

Supplementary Information

## Data Availability

The datasets generated during and/or analysed during the current study are available from the corresponding author on reasonable request.
