# Supplementary material for: Winter warming is ecologically more relevant than summer warming in a cool-temperate grassland
Source: Sci Rep. 2019 Oct 10;9:14632. doi: 10.1038/s41598-019-51221-w (PMC6787088; doi:10.1038/s41598-019-51221-w)
Supplement: Supplementary file 1 — Supplementary Information [file 41598_2019_51221_MOESM1_ESM.pdf]

Supplementary information to:

Winter warming is ecologically more relevant than summer warming in a cool-temperate grassland

Juergen Kreyling, Kerstin Grant, Verena Hammerl, Mohammed A.S. Arfin Khan, Andrey V. Malyshev, Josep Peñuelas, Karin Pritsch, Jordi Sardans, Michael Schloter, Jan Schuerings, Anke Jentsch, Carl Beierkuhnlein

* corresponding author: phone: +49 3834 4204131, juergen.kreyling@uni-greifswald.de

Supplementary information Table 1| Species with preferential occurrence among the warming treatments based on the analysis of indicator species[4]. The relative abundances among the warming treatments for all species with non-random occurrence ($p < 0.05$) are provided. The treatment with the highest relative abundance for each species is in bold type. Plant height, according to Jäger & Rothmaler[54], is used as a surrogate for potential plant biomass production[55] because productivity of a given species cannot be estimated from our biomass harvest as we miss monocultures per species and productivity in a community is biased by competitive effects. No significant preferential occurrence was found for 2009, the pre-treatment year. FG, functional group.

Species	FG	Plant height (cm)	Reference	Summer warming	Winter warming	*p*
Hypochaeris radicata	forb	60	**0.48**	0.16	0.36	0.019
Veronica serpyllifolia	forb	12	**0.79**	0.00	0.21	0.005
Alopecurus pratensis	graminoid	100	0.32	**0.36**	0.32	0.011
Luzula campestris	graminoid	20	0.22	**0.70**	0.09	0.012
Vicia cracca	legume	30-120*	0.08	**0.58**	0.34	0.005
Holcus lanatus	graminoid	100	0.30	0.29	**0.42**	0.007
Arrhenatherum elatius	graminoid	120	0.30	0.27	**0.43**	0.002
Lotus corniculatus	legume	50	0.24	0.28	**0.48**	0.026

* up to 120 cm only by climbing on other species, but produces little biomass per shoot (our observations)

Insight: Species occurring preferentially in the winter-warming treatment consisted of very productive grasses and legumes, the productivity of species occurring preferentially in the summer-warming treatment was inconsistent, and species with preferential occurrence under reference conditions were relatively unproductive forb species.

Supplementary information Figure 1| Environmental conditions in the warming treatments during the four-year study period. a) Snow depth, b) air temperature within the stand (+5 cm), c) soil temperature (-2 cm), and d) soil moisture (-2 to -7 cm) during the study period. During the winter-warming phases (gray blocks), black lines indicate the winter-warming treatment, and gray lines indicate the summer-warming treatment and the reference temperatures. During the summer-warming phases (white blocks), black lines indicate the summer-warming treatment, and gray lines indicate the winter-warming treatment and the reference. Daily means for snow depth (n = 10) and monthly means for air and soil temperatures (n = 10) and soil moisture (n = 30) are displayed.

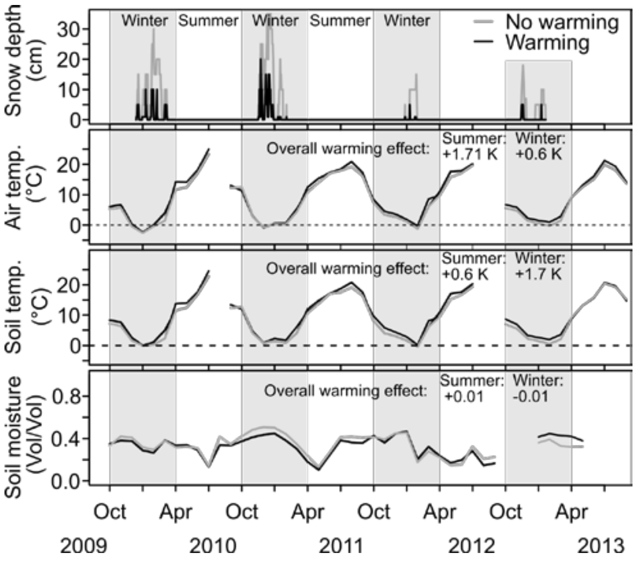

Insight: Four years of winter warming increased soil temperature more than summer warming (+1.7 versus +0.6 °C at -2 cm), and four years of summer warming increased air temperature more than winter warming (+1.7 versus +0.6 °C at +5 cm), probably because the plant tissues partially absorbed the heat generated by the heaters but only a small amount of plant biomass survived the winters.

Supplementary information Figure 2| Potential activities of extracellular enzymes in response to seasonal warming during winter 2011/12. bgls, β-glucosidase; cel, cellobiohydrolase; nag, N-acetyl-glucosaminidase; glr, glucuronidase; pho, acid phosphatase; xyl, xylosidase. Means ± SEMs (n = 10) per sampling date and treatment are displayed.

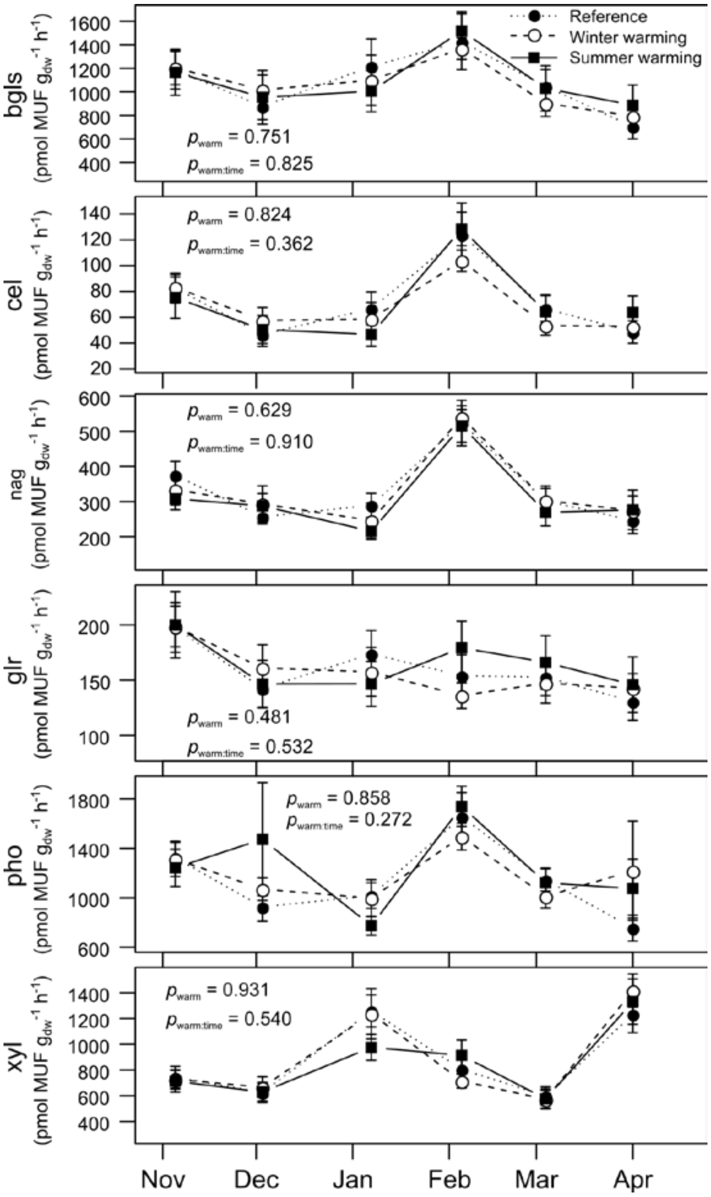

Insight: We found no evidence for winter-warming effects on potential exoenzymatic activity, even though the net effects on ANPP and soil respiration were strong. Potential exoenzymatic activities play important roles in nutrient cycling, and warming increases their production (Davidson & Janssens, 2006) as soil frost decreases it (Sorensen et al. 2016). The 30% increase in winter soil respiration due to the applied warming indicated that soil biotic activity was considerably modified by our winter-warming treatment. The lack of significant warming effects on microbial biomass and potential extracellular enzyme activities may therefore be due to the limited ability to detect such changes given the applied warming.

Methodology:

Potential extracellular enzymatic activity (PEEA) was determined as described by Pritsch *et al.* [56]. Briefly, the enzymatic assays, based on substrates labeled with methylumbelliferone (MUF) (Sigma-Aldrich Chemie, Merck KGaA, Darmstadt, Germany), were prepared in black microplates (VWR International GmbH, Darmstadt, Germany). Substrate saturation concentrations and incubation times for each enzyme were: β-glucosidase (EC 3.2.1.21), 500 µM and 120 min; cellobiohydrolase (EC 3.2.1.91), 400 µM and 120 min; glucuronidase (EC 3.2.1.31), 500 µM and 120 min; xylosidase (EC 3.2.1.37), 500 µM and 60 min; N-acetyl-glucosaminidase (EC 3.2.1.14), 500 µM and 60 min; acid phosphatase (EC 3.1.3.2), 800 µM and 40 min. Autofluorescence of the soil or quenching of the fluorescence signal influenced by the soil was excluded by additional tests using 50 µL of soil suspension and 100 µL of 300 pmol MUF instead of substrate. Fluorescence was measured on a SpectraMax spectrofluorometer (GEMINI EM, Molecular Devices, Silicon Valley, USA) at excitation/emission wavelengths of 365/450 nm. The amounts of MUF released were calculated based on calibration curves and expressed as PEEA (pmol g^{-1} dw h^{-1}). We identified the most relevant enzymes involved in litter decomposition by assessing these enzymatic activities.

Supplementary information Figure 3| Photosynthetic photon flux density (PPFD), snow depth, c) air temperature (+5 cm), soil temperature (-2 cm), and soil moisture (-2 to -7 cm) during the intensive winter sampling campaign of 2011/12. Winter warming was applied throughout the depicted period. Rolling means for snow depth, air temperature, and soil temperature (n = 10) and for soil moisture (n = 30) over 24 h are displayed.

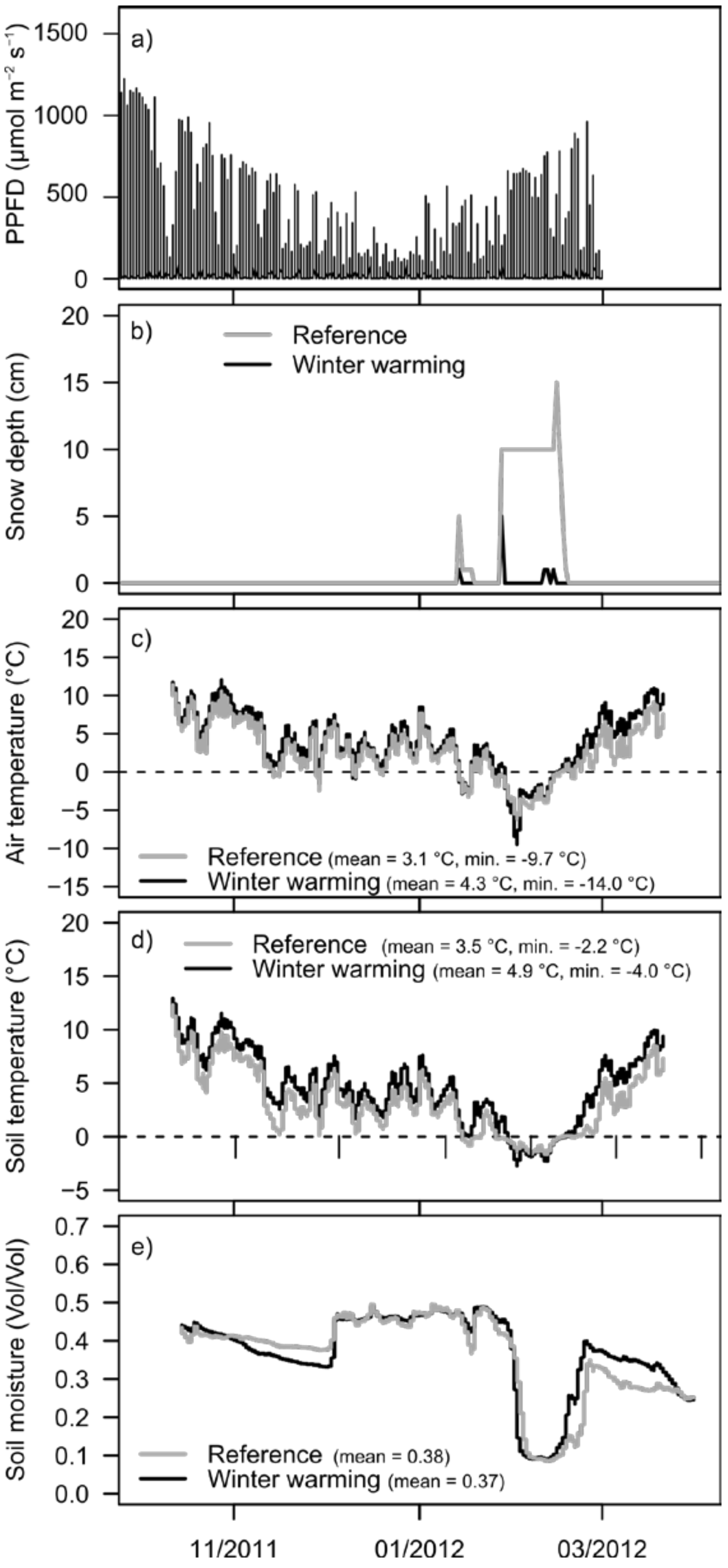

Insight: Winter warming during the intensive winter sampling campaign of 2011/2012 increased air and soil temperatures by 1.2 and 1.4 °C, respectively. Twenty-four soil freeze-thaw cycles (i.e. changes of sign from positive to negative and back in hourly temperature readings) were observed in the warmed plots compared to 46 freeze-thaw cycles in the reference plots. Growing degree hours above 5 °C according to Anderson *et al.*[57] differed only slightly between the warmed and reference plots (915 and 909 h, respectively).

References Supplementary information:

54. E. J. Jäger & W. Rothmaler (eds.). *Exkursionsflora von Deutschland. Gefäßpflanzen: Atlasband* (Springer Spektrum, Berlin, 2013).

55. Moles, A. T. *et al.* Global patterns in plant height. *J Ecol* **97,** 923–932 (2009).

56. Pritsch, K. *et al.* Mycorrhizosphere responsiveness to atmospheric ozone and inoculation with Phytophthora citricola in a phytotron experiment with spruce/beech mixed cultures. *Plant Biology* **7,** 718–727 (2005).

57. Anderson, J. L. & Richardson, E.A., Kesner, C.D. Validation of chill unit and flower bud phenology models for "Montmorency" sour cherry. *Acta Horticulturae* **184,** 71–78 (1986).
